# Polymer Network Evolution Induced by Dual-Crosslinking Architectures in UV-Curable Waterborne Polyurethanes

**DOI:** 10.3390/polym18192354

**Published:** 2026-09-27

**Authors:** Kai-Yen Chin, Jhu-Lin You, Po-Jui Hsieh, Shu-Mei Chang

**Affiliations:** 1 Department of Molecular Science and Engineering, National Taipei University of Technology, Taipei 106, Taiwan; f104gstyle@gmail.com (K.-Y.C.); ray952718@gmail.com (P.-J.H.); 2 Department of Chemical & Materials Engineering, Chung Cheng Institute of Technology, National Defense University, Taoyuan 335, Taiwan; yolin1014001@gmail.com

**Keywords:** waterborne polyurethane, dual-crosslinking, Hydrogen bonding, castor oil, network integrity

## Abstract

Dual-crosslinked waterborne polyurethane (WPU) coatings offer high performance, yet how sequential crosslinking governs the dynamic evolution of internal hydrogen bonding remains poorly understood. Herein, a dual-crosslinking strategy was developed by coupling castor oil (CO) thermal pre-branching with photo-curing monomers of contrasting functionalities: hydroxyl-bearing monofunctional acrylate (2-hydroxyethyl methacrylate, HEMA) and trifunctional acrylate (pentaerythritol triacrylate, PETA). The interplay between covalent crosslinking and physical hydrogen-bonding reorganization was systematically investigated using FTIR carbonyl deconvolution, gel fraction, crosslinking density, and dynamic mechanical analysis. Spectral deconvolution demonstrated that dual-crosslinking reorganizes hard-segment microenvironments rather than merely increasing hydrogen-bond density. Specifically, CO pre-branching facilitated supramolecular association, where CO1 selectively promoted localized, highly ordered microdomains (33.8% strongly bonded carbonyls in WPU-CO1-TA) and CO2 expanded the overall hydrogen-bonded population. DMA further revealed that these topological variations directly dictate segmental relaxation and network integrity across the glass transition. Overall, this preliminary study establishes that tailoring photo-monomer functionality alongside bio-based branching regulates physical hydrogen-bonding redistribution, providing a rational design strategy for advanced dual-crosslinked WPU films.

## 1. Introduction

In recent years, the coating industry has been rapidly moving toward “waterborne,” “solvent-free,” and “green” formulations due to growing environmental consciousness and increasingly strict legislative controls on volatile organic compounds (VOCs) and hazardous air pollutants (HAPs) [1,2,3]. As a promising, eco-friendly material, waterborne polyurethane (WPU) has garnered significant attention by virtue of utilizing water as a dispersion medium instead of organic solvents [4,5]. Currently, it has found extensive applications in various fields, including textiles, leather, wood, plastics, construction, papermaking, automotive manufacturing, and industrial maintenance [5,6,7,8].

Although waterborne polyurethane offers low cost, environmental friendliness, safety, and reliability, achieving successful dispersion of inherently hydrophobic polyurethane in aqueous media requires the strategic incorporation of hydrophilic groups into the PU backbone. A significant drawback is that these residual hydrophilic segments can compromise the material’s water and solvent resistance, drying speed, and mechanical stability [9,10,11].

A direct, simple, and effective approach to enhancing the overall performance of WPU is crosslinking modification [12,13,14]. By incorporating polyfunctional substances into the polymer system, the linear molecular chains transition into a three-dimensional network structure stabilized by covalent or physical interactions. Based on the triggering stage and mechanism, crosslinking methods are primarily categorized into internal crosslinking, self-crosslinking, and external crosslinking. However, a single crosslinking method often exhibits inherent limitations. Therefore, investigating the synergistic influence of multi-crosslinking systems is crucial for achieving balanced performance optimization through phased strategies [3,12,15,16]. Especially in demanding protective applications, achieving a high crosslinking density is vital due to its decisive role in barrier properties and mechanical durability [17,18,19].

Currently reported studies have focused on continually improving corrosion protection and related performance properties through the selection of suitable materials and multiple crosslinking structures [20,21,22,23,24,25,26]. For example, Li et al. [27]. presented a dual-crosslinked WPU with an exceptional corrosion protection efficiency of 99.96% on 304 stainless steel sheets by utilizing a trifunctional crosslinker to establish internal crosslinking. They subsequently blended two types of acrylic monomers—pentaerythritol triacrylate (PETA) and octafluoropentyl methacrylate—with WPU, which was cured under UV irradiation to form a dual-crosslinked network. Another way, Yao et al. [28]. focused on enhancing mechanical and thermal properties by incorporating an amino-rich macromolecular post-crosslinking agent, polyethyleneimine, during dispersion formation into a pre-crosslinked WPU synthesized with an eco-friendly crosslinking agent, castor oil (CO). Even without the addition of the amino-rich macromolecule, this CO-based WPU achieved a gel fraction of over 60% alongside excellent thermal properties.

Despite significant progress in multi-crosslinked WPU systems, existing studies have rarely addressed the stage-wise evolution of intermolecular interactions, particularly the reorganization of hydrogen-bonding environments during sequential crosslinking. Moreover, how different photo-crosslinking architectures regulate hydrogen-bonding organization and the subsequent evolution of polymer networks remains poorly understood. In this work, a rational dual-crosslinking strategy was developed by combining castor oil (CO)-induced branching with two photo-curing monomers possessing distinct functionalities, namely hydroxyl-bearing monofunctional 2-hydroxyethyl methacrylate (HEMA) and trifunctional pentaerythritol triacrylate (PETA). By systematically correlating FTIR carbonyl deconvolution, dynamic mechanical analysis, gel fraction, equilibrium swelling, apparent effective network density, and macroscopic mechanical and thermal performance, this study elucidates how dual-crosslinking architectures govern polymer network evolution and establishes a comprehensive structure–property relationship for environmentally friendly UV-curable waterborne polyurethane coatings.

## 2. Materials and Methods

### 2.1. Synthesis of Dual-Crosslinked WPU

The synthesis pathway and formulations of the WPU emulsions are detailed in Figure 1 and Table A1. The R-value (NCO/OH) for all WPU series was maintained at 1.2. In a typical procedure, poly(ε-caprolactone) diol (PCL-1000, 98%, Mn = 1000 g/mol, Thermo Scientific Chemicals, Waltham, MA, USA) and 2,2-bis(hydroxymethyl)butyric acid (DMBA, 98%, Sigma-Aldrich, St. Louis, MO, USA) were dehydrate d under vacuum at 100 °C under mechanical stirring (55 rpm) for 1 h, followed by the introduction of a nitrogen atmosphere. After the system was cooled to 80 °C, isophorone diisocyanate (IPDI, 99%, Sigma-Aldrich, St. Louis, MO, USA) and dibutyltin dilaurate (DBTDL, Sigma-Aldrich, St. Louis, MO, USA) dissolved in 5 wt% acetone (ECHO Chemical, Miaoli, Taiwan, dehydrated over 4 Å molecular sieves) were added, and the prepolymerization proceeded for 1.5 h. Subsequently, castor oil (CO, First Chemical Co., Ltd., Taipei, Taiwan) was added and allowed to react for 1 h. Next, pentaerythritol triacrylate (PETA, 99%, Sigma-Aldrich, St. Louis, MO, USA) or 2-hydroxyethyl methacrylate (HEMA, 99%, Sigma-Aldrich, St. Louis, MO, USA) was introduced and reacted for 2 h; all acrylate monomers were purified through basic alumina prior to use. After the reaction mixture was cooled to 30 °C, triethylamine (TEA, J.T. Baker, Phillipsburg, NJ, USA) was added as a neutralizer and allowed to react for 30 min to neutralize the carboxyl groups along the polyurethane chains. Deionized water containing 3 wt% (relative to prepolymer weight) of diphenyl(2,4,6-trimethylbenzoyl) phosphine oxide photoinitiator was then introduced under high-speed stirring (2000 rpm) for 15 min to disperse the prepolymer. Finally, acetone was removed using a rotary evaporator to yield a WPU emulsion with a solid content of 40 wt%. The emulsion samples were designated according to their respective formulations. The resulting WPU emulsion was then cast into a mold, air-dried at room temperature for 24 h, and further cured under a 365 nm UV light source (6 mW cm^−2^, Dongxu Material Technology, New Taipei, Taiwan) for 5 min to obtain the final WPU film.

### 2.2. Characterizations

FTIR spectra were obtained in attenuated total reflectance (ATR) mode using a PerkinElmer spectrum One FTIR spectrometer. The average particle size of all WPU dispersions was measured using dynamic light scattering (DLS, Nano Brook 90 Plus, Holtsville, NY, USA) measurement. The mass loss of samples was evaluated by TGA (TG 209 F3, Netzsch, Selb, Germany) at a heating rate of 10 °C/min^−1^ from 30 to 900 °C under N_2_. Dynamic mechanical analysis (DMA) was performed using a DMS 6100 analyzer (TechMax, New Taipei, Taiwan) in film-tension mode. Samples (dimensions: 20 mm × 10 mm × 0.3 mm) were heated from −50 to 150 °C at a constant heating rate of 5 °C min^−1^ under an oscillation frequency of 5 Hz and a force amplitude of 100 mN.

The viscoelastic behavior of the WPU films (cut with 20 × 10 × 0.3 mm^3^) was measured in tensile mode using a DMA (DMS 610, high-tech, HITACHI, Tokyo, Japan). The specimens were heated from −50 to 150 °C at the rate of 2 °C/min with a fixed frequency of 1 Hz. Mechanical testing was performed using a universal testing machine (A2 TYPE, HUNG TA, Taihung, Taiwan) at a crosshead speed of 500 mm/min. Specimens were die-cut into a Type V dumbbell shape (0.9 ± 0.1 mm thickness) in accordance with ASTM D638 [29]. Pencil hardness of the WPU films was evaluated using a B-3084T3 pencil hardness tester under a 765 g load (Yi Chang Enterprise, Kaohsiung, Taiwan), in accordance with ASTM D3363 standards [30].

#### 2.2.1. Chemical Resistance and Gel Fraction

The swelling ratio (%) was evaluated by immersing the WPU films in tetrahydrofuran (THF), N, N-dimethylformamide (DMF), and toluene for 24 h. The initial dry weight of each film (W_0_) was recorded before immersion. After 24 h, the samples were removed, gently blotted with filter paper to remove excess surface solvent, and immediately weighed to obtain the swollen weight (W_1_). The swelling ratio was calculated as follows:

Swelling ratio % = W_1_ − W_0_/W_0_ × 100% (1)

To determine the gel fraction, the toluene-immersed WPU films were subsequently dried in a vacuum oven at 40 °C until a constant weight (W_2_) was reached. The dried insoluble films were weighed, and the gel fraction was calculated using Equation (2):

Gel fraction % = W_2_/W_0_ × 100% (2)

#### 2.2.2. Crosslinking Density

The crosslinking density (V_es_) of the WPU films was determined using the Flory–Rehner equation based on toluene equilibrium swelling results [31]. Film samples (1 cm × 1 cm) were weighed, and their density (*ρ*_c_) was measured using a density balance (AUY220, Shimadzu, Japan). The polymer volume fraction (V_r_) and crosslinking density were calculated using Equations (3) and (4).

The volume frication and crosslinking density was calculated using the following parameters:

(3) $$V_{r}=\frac{\frac{W_{0}}{\rho_{s}}}{\frac{W_{0}}{\rho_{s}}+\frac{\left(W_{1}-W_{0}\right)}{\rho_{c}}}$$

(4) $$V_{es}=\frac{ln(1-V_{r})+V_{r}+xV_{r}^{2}}{V_{0}(0.5V_{r}-V_{r}^{\frac{1}{3}})}$$

where W_0_ is the initial dry mass of the film, W_1_ is the swollen mass at equilibrium, *ρ*_s_ is the density of toluene (0.867 g/cm^3^), χ is the polymer–solvent interaction parameter (χ = 0.454), and V_0_ is the molar volume of toluene 106.3 cm^3^·mol^−1^.

## 3. Results and Discussion

### 3.1. Construction and Structural Characterization of Dual-Crosslinked Waterborne Polyurethane Networks

To investigate how different crosslinking architectures influence the subsequent evolution of polymer networks, the successful construction of the designed dual-crosslinked WPU systems was first verified at the molecular level. As illustrated in Figure 1, bio-based castor oil (CO) was incorporated during polyurethane synthesis to introduce internal branching points within the polymer backbone, whereas photo-reactive acrylate components with different functionalities were subsequently introduced to generate a secondary covalent network through UV-induced photopolymerization. This sequential design enables the individual and cooperative contributions of bio-based branching and photo-crosslinking to be systematically distinguished.

FTIR spectroscopy was first employed to confirm the successful formation of the polyurethane backbone. As shown in Figure 2a, all samples exhibited a broad absorption band at 3100–3500 cm^−1^, attributed to the overlapping of N–H from urethane groups and potentially physically adsorbed water. Concurrently, the characteristic urethane carbonyl C=O stretching band appeared near 1700 cm^−1^, confirming the successful formation of urethane linkages [32]. The dual absorption peaks at 2920 and 2850 cm^−1^ correspond to the asymmetric and symmetric C–H stretching vibrations of aliphatic methylene segments, while the band at 1450 cm^−1^ is assigned to aliphatic C–H bending deformations. Additionally, the diagnostic doublet observed at 1385 and 1365 cm^−1^ is attributed to the gem-dimethyl bending splitting of the cycloaliphatic ring originating from IPDI. Furthermore, the peak centered at 1530 cm^−1^ is attributed to the coupled C–N stretching and N–H bending (Amide II) of the urethane framework [33,34,35], whereas the band at 1310 cm^−1^ is associated with N–H in-plane bending. The absorption peak at 1225 cm^−1^ reflects C–O stretching within the urethane linkages (–NH–COO–), and the bands at 1150, 1030, and 770 cm^−1^ are characteristic of ester/ether C–O–C stretching and skeletal vibrations [36,37,38].

More importantly, no detectable absorption corresponding to the isocyanate (–NCO) group was observed at approximately 2270 cm^−1^, indicating that the isocyanate groups had been effectively consumed during polyurethane synthesis.

To further verify the formation of the photo-crosslinked network, the FTIR spectra before and after UV irradiation were compared within the fingerprint region (1000–650 cm^−1^). As presented in Figure 2b, the characteristic acrylate C=C–H bending vibration at approximately 810 cm^−1^ was clearly observed in the WPU-MA, WPU-TA, WPU-CO1-TA, and WPU-CO2-TA samples prior to UV exposure [39]. However, in complex formulations containing polyols, bio-based oils, isocyanates, and reactive diluents, the 800 to 850 cm^−1^ region is spectrally congested due to various overlapping bending and skeletal vibrations. Specifically, this window encompasses C–CH_3_ rocking modes from methacrylate groups, skeletal deformations of the cycloaliphatic ring originating from IPDI, and out-of-plane skeletal deformations of the newly formed urethane groups (–NH–CO–O–). Following UV irradiation, the intensity of the diagnostic acrylate band at ~810 cm^−1^ diminished substantially, whereas residual background absorbance from the stable polymer skeleton persisted. This selective attenuation is attributed to the effective consumption of acrylate double bonds during free-radical photopolymerization. These observations confirm that the prepolymers were successfully converted into an interconnected photo-crosslinked network upon film formation.

The influence of molecular architecture on the colloidal organization of the WPU dispersions was further evaluated by DLS. As shown in Figure 3, the neat WPU exhibited an average particle size of 23.9 nm and appeared as a relatively transparent dispersion with a characteristic bluish tint. The introduction of the photo-reactive components alone caused only minor changes, with average particle sizes of 25.1 nm for WPU-MA and 25.4 nm for WPU-TA, indicating that these structures did not substantially alter the colloidal dimensions of the WPU dispersions before UV curing.

In contrast, castor-oil incorporation produced a pronounced increase in particle size. The average diameters of WPU-CO1 and WPU-CO2 increased to 100.6 and 148.9 nm, respectively, accompanied by a gradual transition from a translucent bluish dispersion to a more opaque, milky appearance. This behavior is reasonably attributed to the long hydrophobic fatty-acid chains and multifunctional structure of castor oil, which enhanced hydrophobic association within the dispersed particles and promoted the formation of larger latex domains [40,41].

The larger particle size of WPU-CO2 further indicates that increasing the castor-oil content intensified this association. The combined CO/PETA systems exhibited even greater particle dimensions, reaching 175.8 nm for WPU-CO1-TA and 179.1 nm for WPU-CO2-TA. Since the DLS measurements were performed before UV irradiation, these increases do not directly reflect the formation of a photo-crosslinked network. Rather, they indicate that the simultaneous presence of castor-oil-derived branching structures and multifunctional photo-reactive components modified the pre-curing molecular organization and hydrophobic association of the latex particles. Nevertheless, all formulations remained stable waterborne dispersions with nanoscale particle dimensions suitable for subsequent film casting and UV curing.

Overall, the spectroscopic and colloidal results confirm that the designed WPU systems were successfully constructed through two complementary structural elements: castor-oil-derived covalent branching formed during polyurethane synthesis, and a secondary acrylate network generated through post-film UV curing. This sequential design is expected to reorganize the polymer network through distinct crosslinking architectures rather than merely increasing the total number of crosslinking sites. The following section therefore examines how these architectures redistribute the urethane hydrogen-bonding environments and drive subsequently polymer network evolution.

### 3.2. Crosslinking Architecture-Driven Hydrogen-Bonding Organization

Although the FTIR spectra presented in Section 3.1 confirmed the successful formation of the modified WPU systems, they do not directly distinguish the hydrogen-bonding environments within the polyurethane network. Therefore, the carbonyl stretching region was further deconvoluted to quantitatively evaluate the distribution of free carbonyl, hydrogen-bonded carbonyl, and strongly hydrogen-bonded carbonyl species resulting from different crosslinking architectures.

As illustrated in Figure 4, the carbonyl absorption band was resolved into three characteristic components centered at approximately 1730 cm^−1^, 1710 cm^−1^, and 1690 cm^−1^, corresponding to free carbonyl, hydrogen-bonded carbonyl, and strongly hydrogen-bonded carbonyl species, respectively [42,43]. Additionally, to account for potential interference from water bending vibrations, a sub-band near ~1640 cm^−1^ was also included in the deconvolution. The calculated hydrogen-bonding parameters are summarized in Table 1.

Neat WPU showed an overall hydrogen-bonded carbonyl fraction of 56.9%, with strongly hydrogen-bonded carbonyls accounting for 16.0%. This indicates that while more than half of the urethane carbonyl groups participated in hydrogen bonding, only a limited proportion assembled into highly ordered domains. Upon incorporating castor oil, the overall hydrogen-bonded fraction increased significantly to 81.9% for WPU-CO1 and 85.8% for WPU-CO2, demonstrating that the branched bio-based structure promoted hydrogen-bond formation within the polyurethane network. However, despite exhibiting nearly identical overall hydrogen-bonded fractions, WPU-CO2 possessed a markedly higher strongly hydrogen-bonded carbonyl fraction (58.1%) than WPU-CO1 (51.1%). This divergence suggests that the two castor-oil-derived architectures influenced hydrogen-bond organization differently: CO2 preferentially promoted the formation of localized ordered hydrogen-bond domains, whereas CO1 predominantly increased the overall population of hydrogen-bonded carbonyl groups without substantially enhancing local ordering.

A similar tendency was observed after incorporating the photo-crosslinking structures. Compared with WPU-MA, which exhibited an overall hydrogen-bonded carbonyl fraction of 84.1% and a strongly hydrogen-bonded carbonyl fraction of 41.7%, WPU-TA showed a higher overall hydrogen-bonded carbonyl fraction (89.9%) but a lower proportion of strongly hydrogen-bonded carbonyl species (37.9%). These opposite trends clearly indicate that the multifunctional TA-derived network did not simply increase the extent of hydrogen bonding. Instead, the higher crosslink density of TA predominantly expanded the diffuse hydrogen-bonded population while constraining the assembly of locally ordered hard-segment domains. In contrast, the MA-derived network permitted greater conformational flexibility, thereby facilitating the development of highly ordered hydrogen-bonding environments.

The effect of hydrogen-bonding reorganization became even more pronounced in the dual-crosslinked systems. WPU-CO1-TA exhibited an overall hydrogen-bonded carbonyl fraction of 82.2%, while simultaneously achieving the highest strongly hydrogen-bonded carbonyl fraction (63.3%) among all formulations. In contrast, WPU-CO2-TA showed an overall hydrogen-bonded fraction of 70.4%, yet maintained the second-highest strongly bonded fraction at 62.7%. If the dual-crosslinking strategy had merely increased the total extent of hydrogen bonding indiscriminately, these two parameters would be expected to rise in tandem. Instead, these divergent trends demonstrate that the dual-crosslinked architectures reorganized the hydrogen-bonding environments rather than simply increasing hydrogen-bond density. Specifically, WPU-CO1-TA contained a larger proportion of localized, strongly hydrogen-bonded domains coexisting with relatively free carbonyl groups, resulting in a heterogeneous hydrogen-bonding organization. Conversely, WPU-CO2-TA developed a more homogeneously hydrogen-bonded network, in which a larger proportion of carbonyl groups participated in hydrogen bonding while maintaining sufficient strongly bonded domains to preserve network integrity. Overall, the carbonyl deconvolution analysis demonstrates that the principal role of castor-oil branching and photo-crosslinking is not merely to increase hydrogen bonding, but to regulate the spatial organization of hydrogen-bonding environments within the polyurethane hard segments.

### 3.3. Crosslinking Architecture Governs Segmental Relaxation and Network Integrity

To further investigate the influence of different crosslinking architectures on the segmental mobility of WPU coatings, DMA was performed by analyzing the temperature-dependent tan δ curves of the prepared samples. Unlike the storage modulus (E′), which primarily reflects the stiffness of the material, the tan δ response provides direct information on chain relaxation behavior (Figure 5a,b). In general, the position and shape of the tan δ peak are closely related to the glass transition and the distribution of molecular relaxation environments, whereas peak broadening or the appearance of shoulders generally indicates heterogeneous chain mobility arising from different degrees of molecular confinement [44]. Therefore, the DMA results were further correlated with the hydrogen-bonding environments identified by FTIR carbonyl deconvolution.

Among the single-crosslinked systems, WPU-MA exhibited a sharp and well-defined relaxation peak centered at approximately 48 °C, indicating that most polymer chains experienced relatively uniform molecular constraints during thermal relaxation. In contrast, WPU-TA displayed a noticeably broader relaxation profile with an obvious shoulder and a significantly higher relaxation temperature of approximately 105 °C, suggesting the coexistence of multiple chain relaxation environments. This difference agrees well with the FTIR results discussed in Section 3.2. This difference agrees well with the FTIR results discussed in Section 3.2. WPU-TA exhibited a higher hydrogen-bonding index (HBI = 8.9), while WPU-MA showed a larger proportion of strongly hydrogen-bonded carbonyl groups (41.7% versus 37.9% for WPU-TA). This indicates that the multifunctional PETA-derived network redistributed hydrogen-bonding interactions toward a more extensive, diffuse network throughout the cross-linked matrix, rather than allowing chains to assemble into highly ordered hard-segment domains.

Consequently, polymer chains experienced different levels of molecular confinement, giving rise to the broadened relaxation profile observed for WPU-TA. A similar tendency was observed for the dual-crosslinked systems. Although both WPU-CO1-TA and WPU-CO2-TA incorporated castor-oil-derived branching together with multifunctional photo-crosslinking, their relaxation behaviors differed considerably. WPU-CO1-TA exhibited a relaxation peak at approximately 85 °C, together with the highest proportion of strongly hydrogen-bonded carbonyl groups (63.3%) and an HBI of 4.6. These results suggest that the polymer network contained highly constrained hydrogen-bond-rich domains coexisting with relatively free chain regions, resulting in heterogeneous relaxation behavior.

Moreover, the lower relaxation temperature compared with WPU-TA indicates that the long aliphatic chains introduced by castor oil partially compensated for the chain-confinement effect arising from multifunctional photo-crosslinking. In comparison, WPU-CO2-TA exhibited a more concentrated relaxation peak centered at approximately 95 °C, without the pronounced multiple-relaxation characteristics observed for WPU-CO1-TA. FTIR analysis further revealed that WPU-CO2-TA possessed the HBI (2.4) and the second-highest strongly hydrogen-bonded carbonyl fraction (62.7%), indicating a more uniformly distributed hydrogen-bonding network throughout the polymer matrix. Consequently, most polymer chains experienced comparable molecular constraints, leading to a more homogeneous relaxation response.

Interestingly, the relaxation behavior did not simply follow the overall extent of hydrogen bonding but rather reflected how hydrogen-bonding interactions were spatially organized within the polymer network. While WPU-CO1-TA contained the highest proportion of strongly hydrogen-bonded carbonyl groups, the coexistence of strongly associated and weakly associated chain regions resulted in a broader relaxation distribution. Conversely, the more balanced hydrogen-bonding environment established in WPU-CO2-TA produced a comparatively uniform segmental relaxation behavior despite its lower overall hydrogen-bonding level. Overall, the DMA results demonstrate that the segmental relaxation behavior of WPU coatings is governed not only by photo-crosslinking but also by the distribution of hydrogen-bonding interactions and the flexible branching introduced by castor oil.

### 3.4. Evaluation of Network Integrity

The network integrity of the UV-cured coatings was further assessed through gel fraction, equilibrium swelling, and apparent effective network density analyses using toluene as the swelling solvent. Toluene provides sufficient swelling while preserving the integrity of the crosslinked network, making it suitable for quantitative evaluation using the Flory–Rehner equation [31]. For comparison, additional swelling measurements were also performed using THF and DMF. However, because these solvents possess stronger solvent–polymer interactions with polyurethane and may significantly influence hydrogen-bonding associations and physical network structures, their swelling behavior cannot be directly correlated with the effective crosslinked network to the same extent as toluene. Therefore, the discussion of network evolution and the calculation of apparent effective network density in this study are primarily based on the toluene results, whereas the THF and DMF data are presented for comparative reference only.

The gel fraction measurements clearly demonstrate the progressive development of an insoluble polymer network with increasing crosslinking complexity, as shown in Figure 6a. Neat WPU exhibited an almost negligible gel fraction in toluene, indicating that the coating relied primarily on physical hydrogen bonding and chain entanglement and therefore lacked a stable chemically crosslinked network. Incorporation of castor oil alone increased the gel fraction to 86.5 ± 1.6% for WPU-CO1 and 88.2 ± 1% for WPU-CO2, suggesting that the branched castor-oil structure contributed to improved network stability but was still insufficient to establish a highly integrated polymer network. Following UV curing, the gel fraction further increased to 91.9 ± 0.7% for WPU-MA and 92.1 ± 0.7% for WPU-TA, confirming the formation of stable insoluble networks through photochemical crosslinking. More importantly, combining castor-oil branching with multifunctional TA photo-crosslinking produced the highest gel fractions, reaching 93.8 ± 0.5% for WPU-CO1-TA and 95.1 ± 0.5% for WPU-CO2-TA.

The continuous increase in gel fraction indicates that the dual-crosslinking strategy effectively increased the proportion of insoluble network chains and promoted the formation of a more integrated crosslinked structure. The equilibrium swelling behavior provides complementary evidence for the evolution of network integrity, as shown in Figure 6b. As the polymer network became progressively more integrated, solvent uptake was correspondingly suppressed. The equilibrium swelling ratio in toluene decreased from 73% for WPU-CO1 and 64.8% for WPU-CO2 to 65.7% for WPU-MA and 59.7% for WPU-TA, before reaching 58.8% for WPU-CO1-TA and only 53.6% for WPU-CO2-TA.

Furthermore, the evolution of network integrity was quantitatively confirmed by the apparent effective network density calculated from the equilibrium swelling data using the Flory–Rehner equation, as shown in Table 2. The apparent effective network density increased from only 260.1 ± 17.2 mol m^−3^ for WPU-CO1 and 332 ± 20.4 mol m^−3^ for WPU-CO2 to 2001 ± 110 mol m^−3^ for WPU-MA and 2189± 117 mol m^−3^ for WPU-TA, demonstrating that UV curing fundamentally transformed the polyurethane into a chemically crosslinked network. Incorporation of castor oil into the TA-crosslinked system further increased the apparent effective network density to 2230 ± 99 mol m^−3^ for WPU-CO1-TA, while WPU-CO2-TA exhibited the highest value of 2652 ± 115 mol m^−3^ among all investigated formulations. Notably, these quantitative results closely parallel the trends observed in gel fraction and equilibrium swelling, providing mutually consistent evidence that the CO2/TA dual-crosslinked architecture generated the most structurally integrated polymer network.

More importantly, the network evolution revealed by these solvent-based analyses is in excellent agreement with the molecular-level characterization presented in the previous sections. FTIR carbonyl deconvolution demonstrated that the CO2/TA system possessed the highest proportion of hydrogen-bonded carbonyl species, while DMA revealed more restricted segmental relaxation compared with the other formulations. The higher gel fraction, lower equilibrium swelling, and highest apparent effective network density observed here provide direct macroscopic evidence that these molecular interactions were successfully translated into a more continuous and mechanically stable polymer network. The excellent consistency among FTIR, DMA, and solvent-based analyses therefore provides the structural basis for the mechanical and thermal performance discussed in the Section 3.5.

### 3.5. Mechanical and Thermal Performance

To verify whether this structural enhancement translates into superior performance in practical engineering applications, researchers systematically evaluated the tensile behavior, fracture morphology, surface hardness, and thermal stability of the UV-cured coatings. The tensile properties of the UV-cured coatings are summarized in Table 3, while the representative stress–strain curves are presented in Figure 7a.

Following UV curing, distinct differences in mechanical performance were observed between the MA- and TA-crosslinked systems. WPU-MA exhibited a tensile strength of 10.2 MPa, an elongation at break of 102.8%, and a pencil hardness of 4H, indicating that the difunctional MA structure provided only limited reinforcement of the polymer network. In contrast, WPU-TA exhibited substantially improved mechanical performance, with the tensile strength increasing to 23.2 MPa, the elongation at break increasing to 221.3%, and the surface hardness increasing to 5H. These improvements indicate that the multifunctional TA architecture generated a more effective photo-crosslinked network capable of enhancing load-bearing capability while maintaining satisfactory ductility. This behavior is consistent with the higher apparent effective network density and lower equilibrium swelling ratio discussed in the Section 3.4, confirming that the improved network integrity effectively promoted stress transfer throughout the polymer matrix.

Further enhancement was achieved through the dual-crosslinking strategy combining castor-oil branching with TA photo-crosslinking. The tensile strength increased to 30.4 MPa for WPU-CO1-TA and reached 36.0 MPa for WPU-CO2-TA, representing the highest mechanical strength among all investigated formulations. Simultaneously, the elongation at break remained relatively high at 325.5% for WPU-CO1-TA and 288.8% for WPU-CO2-TA, while the pencil hardness further increased to 6H for WPU-CO2-TA. Although the slight reduction in elongation observed for WPU-CO2-TA suggests increased restriction of chain deformation, the simultaneous enhancement in tensile strength and surface hardness indicates that the optimized dual-crosslinking architecture substantially improved the efficiency of stress transfer without causing excessive embrittlement. More importantly, these mechanical trends closely parallel the molecular- and network-level observations established in the previous sections, including the higher hydrogen-bonded carbonyl fraction, more restricted segmental relaxation, higher gel fraction, lower equilibrium swelling ratio, and higher apparent effective network density. Collectively, these results demonstrate that the synergistic dual-crosslinking architecture effectively transformed molecular-level interactions into superior macroscopic mechanical performance.

The fracture morphologies shown in Figure 8 further support the tensile results. Compared with WPU-TA, both dual-crosslinked formulations exhibited more continuous and denser fracture surfaces, while WPU-CO2-TA displayed a noticeably rougher fracture morphology with more pronounced deformation features after tensile failure. These observations suggest that greater energy was required during crack propagation, consistent with its superior tensile strength and higher network integrity [19]. The fracture morphology not only reveals an increase in crosslink density but also provides direct evidence that the optimized network structure facilitates more effective stress distribution within the polymer matrix during mechanical deformation. The excellent agreement between the fracture morphology and the tensile results therefore provides additional support for the formation of a mechanically robust and highly integrated polymer network.

The thermal degradation behaviors of the coatings are presented in Figure 7b,c. All formulations exhibited the characteristic two-stage thermal decomposition behavior of polyurethane systems, with the principal degradation occurring between approximately 250 and 450 °C. Relative to pristine WPU, both the castor-oil-modified and photo-crosslinked systems exhibited delayed thermal degradation behavior, indicating that the introduction of branched structures and photo-crosslinked networks effectively enhanced the thermal resistance of the polymer matrix. Among the UV-cured coatings, the dual-crosslinked WPU-CO1-TA and WPU-CO2-TA systems exhibited slightly delayed degradation behavior compared with the corresponding single-crosslinked formulations, suggesting that the more integrated polymer network effectively restricted thermally induced chain scission during heating. Although the improvement in degradation temperature was moderate, the thermal behavior consistently followed the same trend observed in the solvent resistance and tensile analyses, indicating that the enhanced network integrity contributed not only to improved mechanical robustness but also to increased resistance against thermal degradation.

Overall, these results clearly demonstrate that the dual-crosslinking strategy not only improves the integrity of the polymer network but also translates these structural advantages into enhanced mechanical performance and thermal stability. The good agreement among the molecular, network, and macroscopic characterizations provides a comprehensive understanding of the structure–property relationship in the developed UV-cured coatings.

## 4. Conclusions

A dual-crosslinking strategy integrating castor oil branching with pentaerythritol triacrylate photo-crosslinking was successfully developed to fabricate environmentally friendly UV-curable waterborne polyurethane coatings with improved network integrity and overall performance. The synergistic effect of castor oil and TA regulated the hydrogen-bonding environment, restricted segmental relaxation, and promoted the formation of a more integrated polymer network, which was supported by the increased gel fraction, reduced equilibrium swelling ratio, and higher apparent effective network density. Consequently, the dual-crosslinked coatings exhibited superior mechanical properties, fracture resistance, surface hardness, and thermal stability. Among all formulations, WPU-CO2-TA exhibited the best overall performance, achieving a tensile strength of 35.949 MPa, an elongation at break of 288.811%, and a pencil hardness of 6H. Overall, this preliminary study provides foundational insights into the structure–property relationships of dual-cross-linked polyurethane networks. Although FTIR and other analyses verified successful photo-polymerization and network densification, further investigations utilizing advanced spectroscopic techniques will be valuable to elucidate the subtle molecular rearrangements and localized conversion of acrylate double bonds. Building upon these initial findings, ongoing work will focus on fine-tuning dual-crosslinking kinetics and exploring long-term environmental durability for advanced protective coating applications.

## Figures and Tables

**Figure 1:**
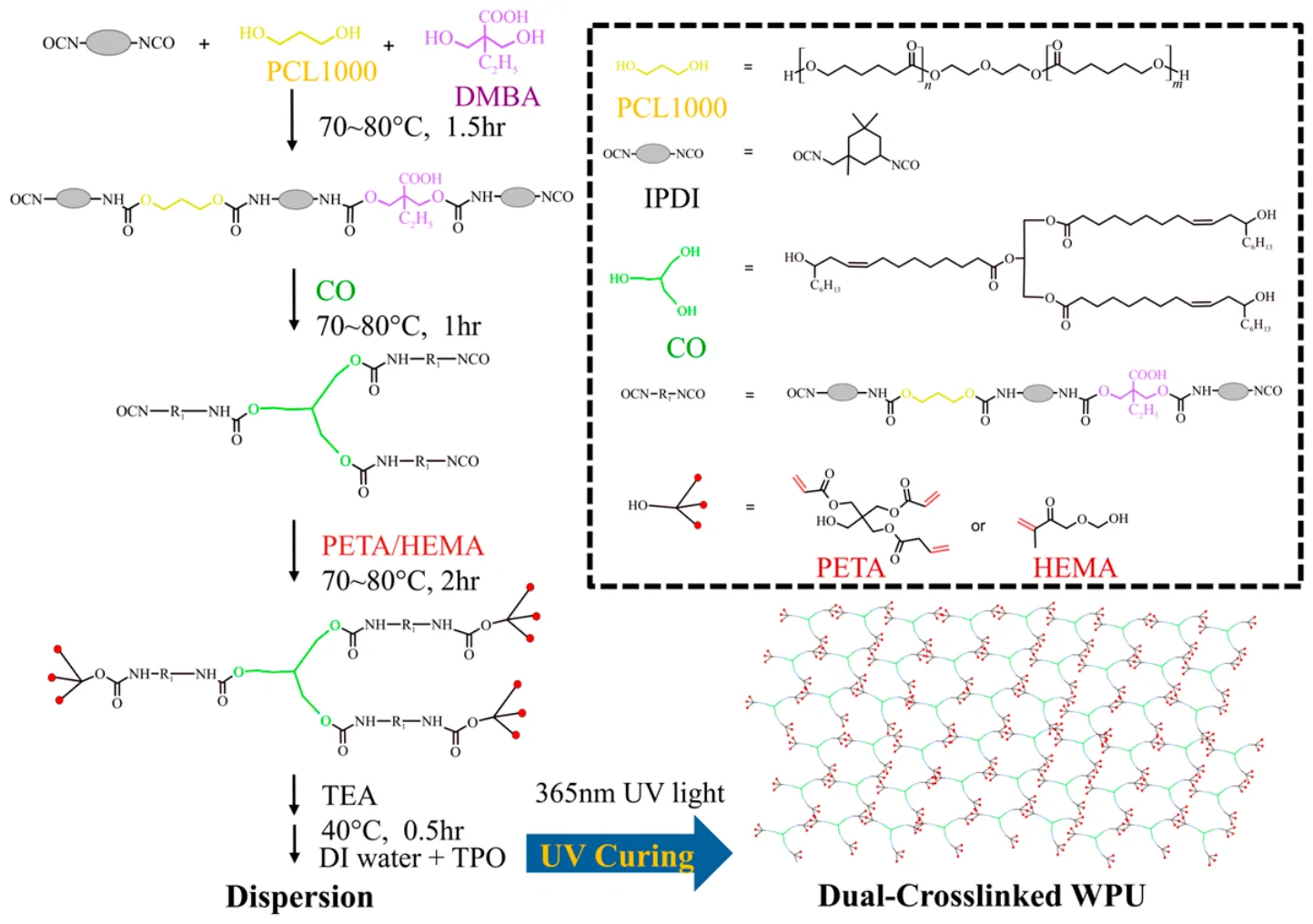
Synthesis pathway of dual-crosslinked WPU.

**Figure 2:**
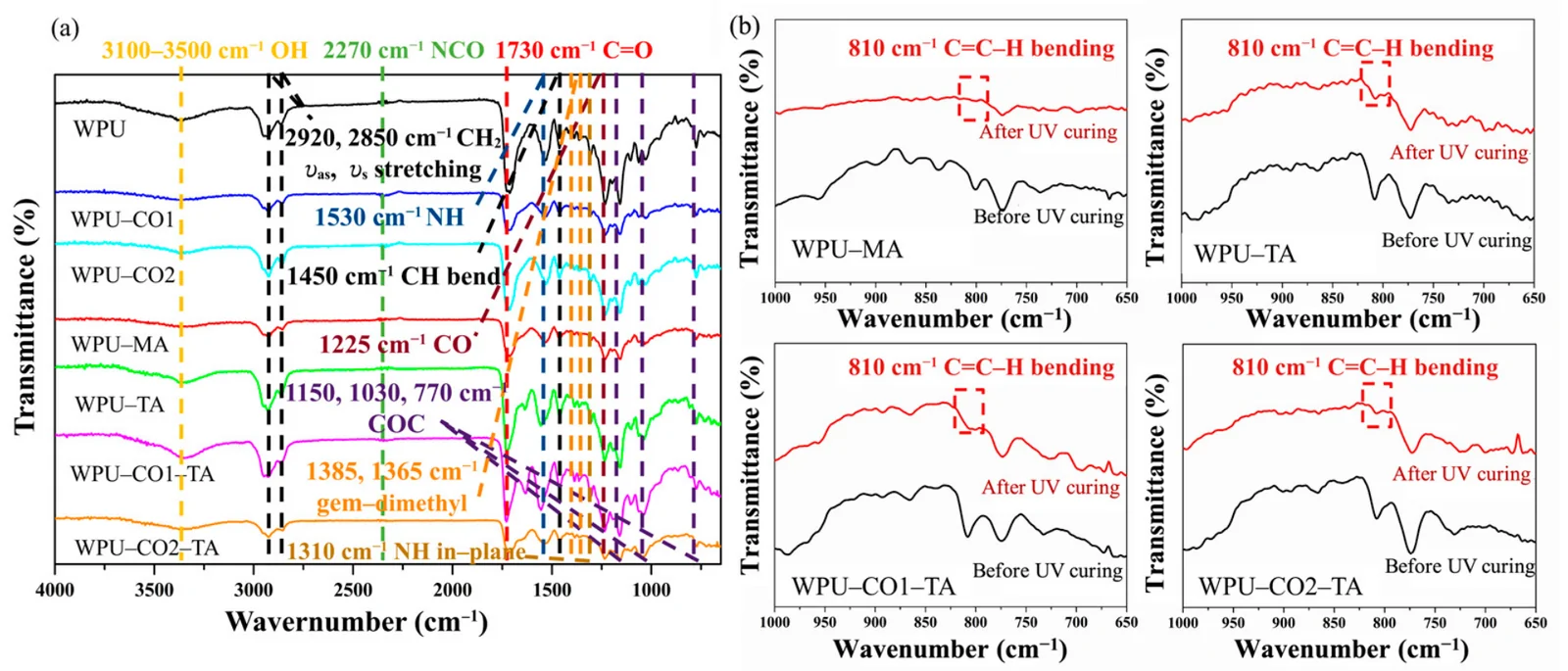
The FTIR spectra of (**a**) WPU film and (**b**) before and after UV curing.

**Figure 3:**
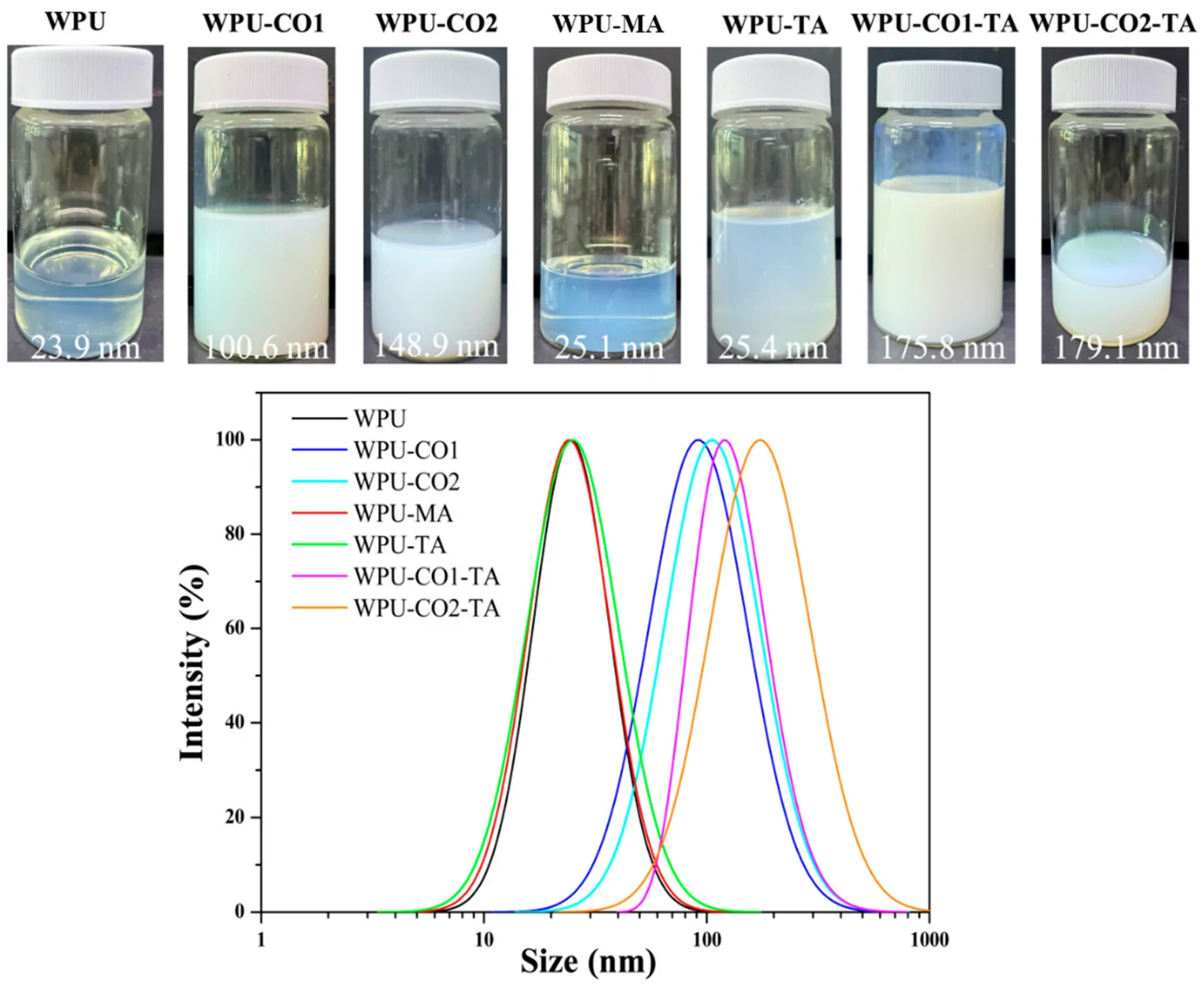
Visual appearance and particle size data of WPU emulsions.

**Figure 4:**
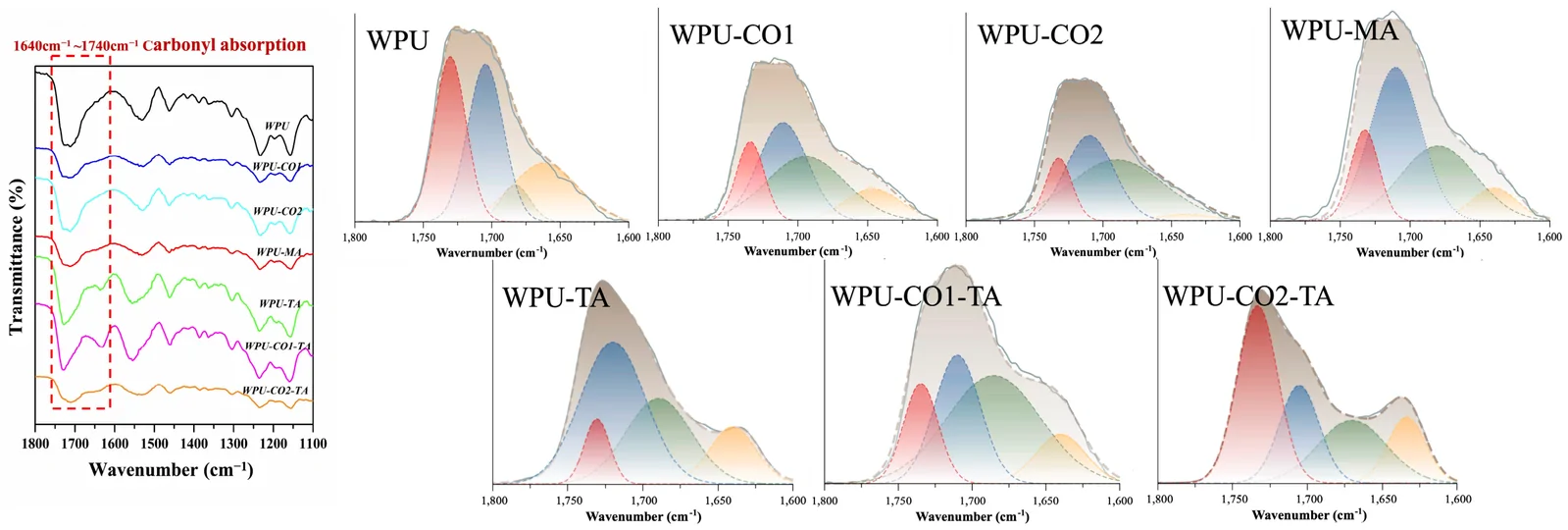
Deconvoluted FTIR carbonyl stretching spectra (1600–1780 cm^−1^) of various WPU films. Peak assignments: water bending (yellow peak, ~1635–1660 cm^−1^), strongly hydrogen-bonded carbonyls (green peak, ~1680–1690 cm^−1^), hydrogen-bonded carbonyls (blue peak, ~1705–1711 cm^−1^), and free carbonyls (red peak, ~17,328–1735 cm^−1^).

**Figure 5:**
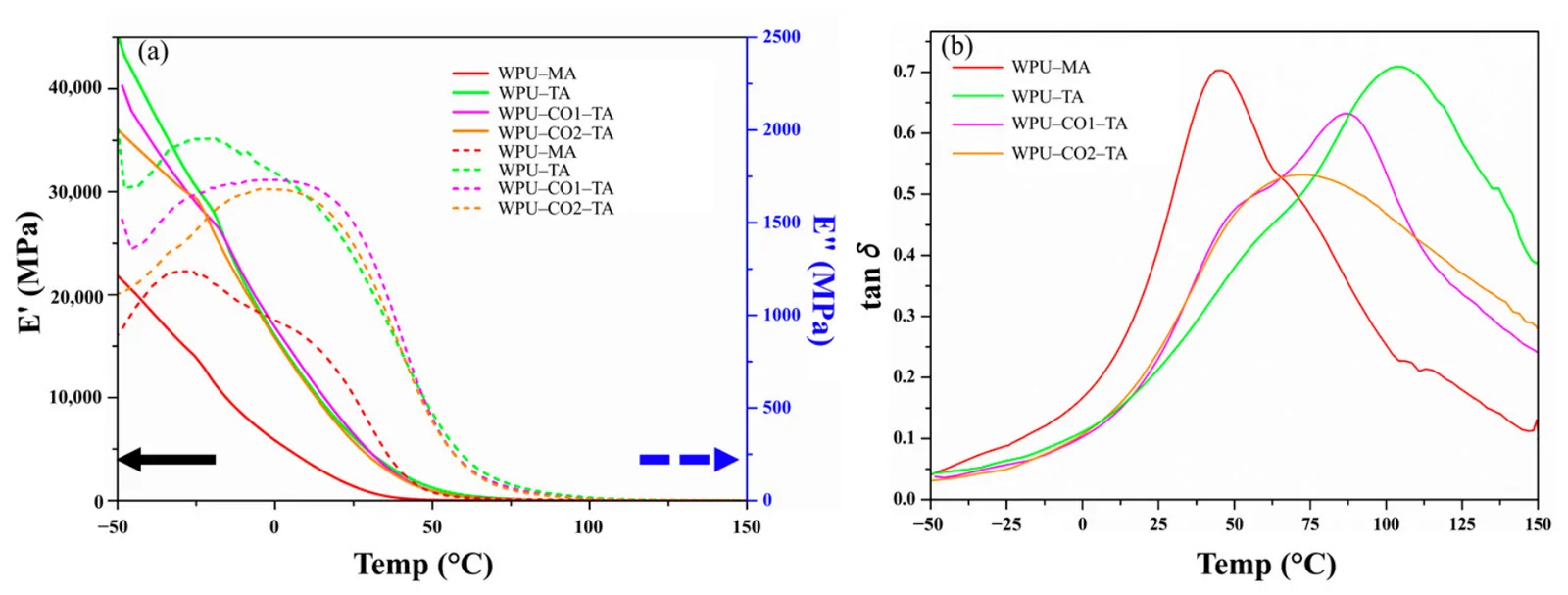
DMA curves of (**a**) storage modulus (E′) and loss modulus (E″), and (**b**) tan δ for synthesized WP films.

**Figure 6:**
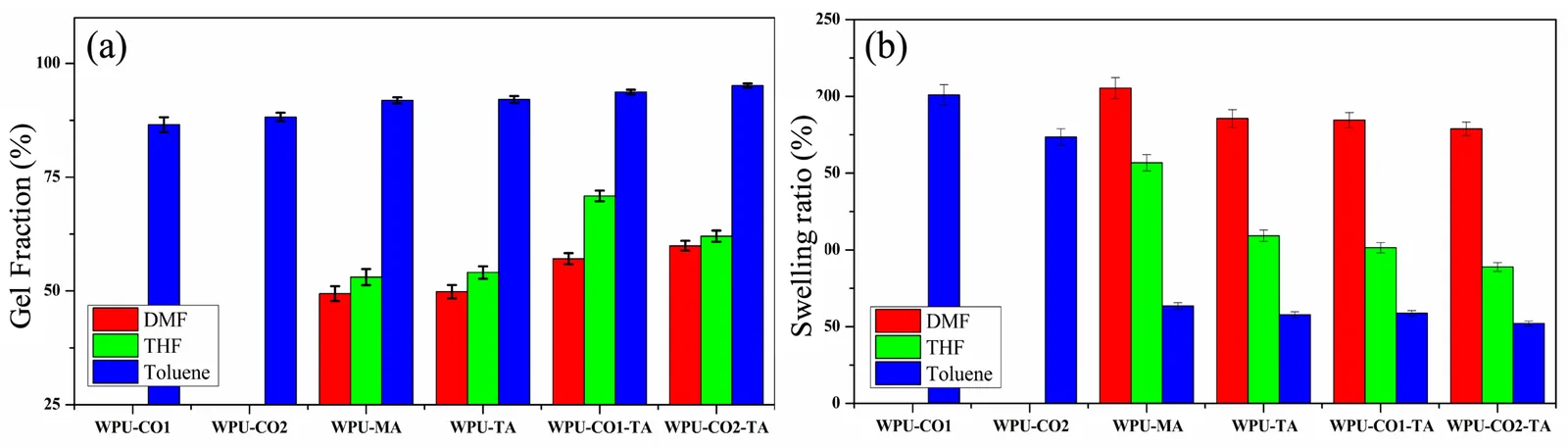
(**a**) Gel fraction and (**b**) swelling ratio of synthesized WP films.

**Figure 7:**
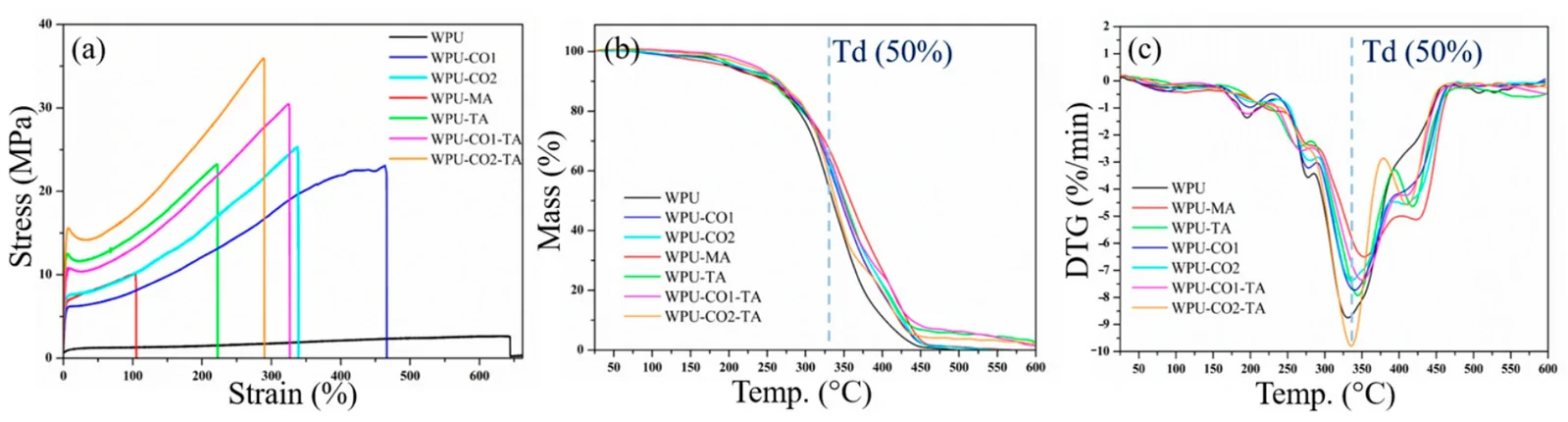
(**a**) Stress–strain curves, (**b**) TGA and (**c**) DTG profiles of the synthesized WPU films.

**Figure 8:**
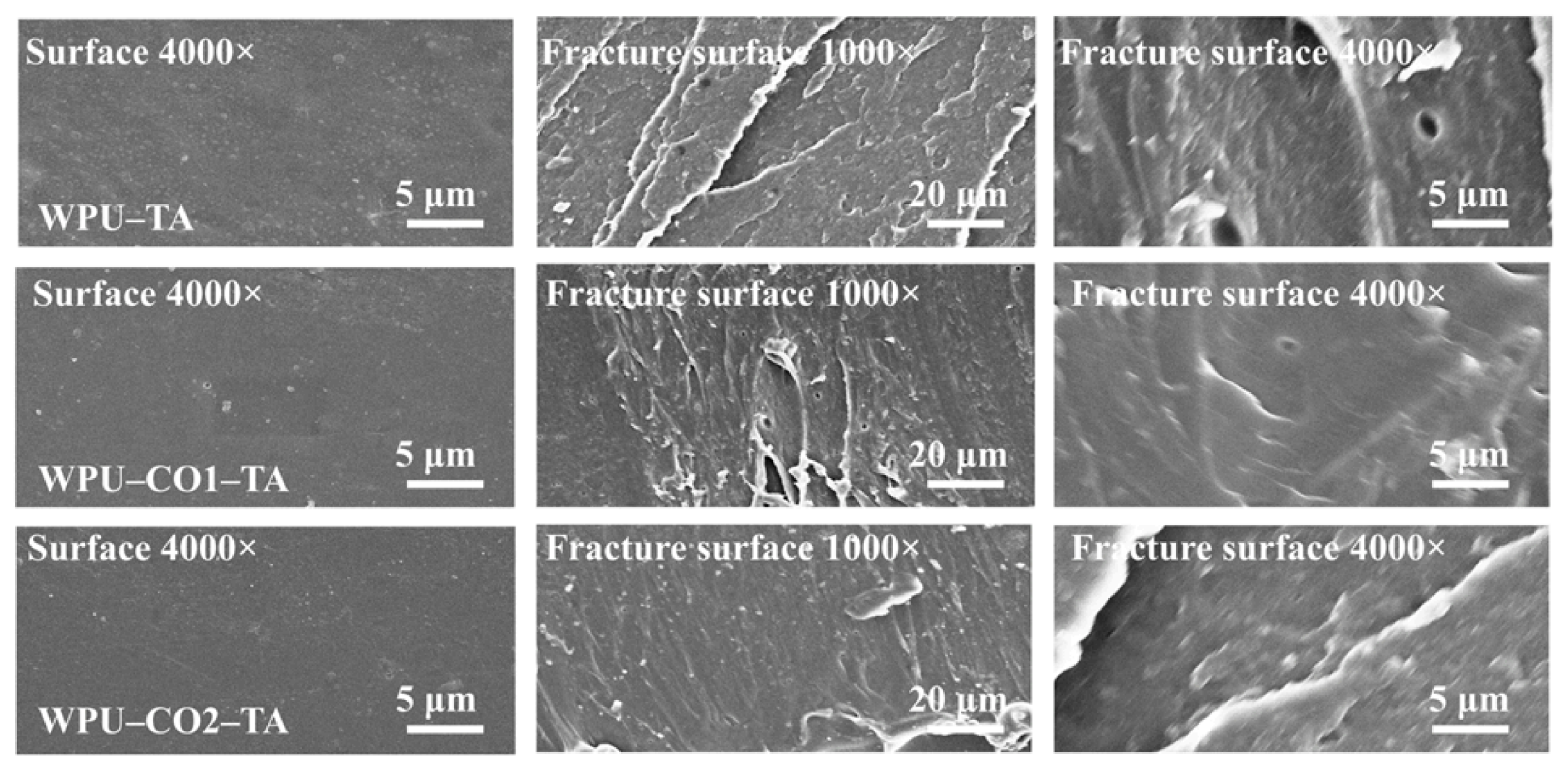
Surface and fracture SEM micrographs of synthesized WPU films.

**Table 1:** Hydrogen-bonding parameters derived from deconvoluted carbonyl FTIR spectra.

Sample	A_R_ Free C=O (%)	A_B_ H-Bond C=O (%)	A_G_ Ordered/Strong H-Bond (%)	A_Y_ Water Bending (%)	* HBI	H-Bonded C=O Fraction (%)	Strongly H-Bonded C=O Fraction (%)
WPU	33.3	35.7	6.8	24.3	1.3	56.9	16
WPU-CO1	15.8	34.9	36.5	12.8	4.5	81.9	51.1
WPU-CO2	13.8	34.9	48.4	2.9	6.0	85.8	58.1
WPU-MA	14.6	44.8	32.1	8.5	5.3	84.1	41.7
WPU-TA	8.7	48	29.3	14	8.9	89.9	37.9
WPU-CO1-TA	15.8	26.8	46.2	11.2	4.6	82.2	63.3
WPU-CO2-TA	23.4	20.8	34.9	20.9	2.4	70.4	62.7

* Hydrogen Bond Index, A_Y_, A_G_, A_B_, A_R_: Area for Yellow, Green, Blue and Red peak.

**Table 2:** Gel fraction, swelling ratio, and crosslinking density parameters of the synthesized WPU films.

Sample	* G_DMF_ (%)	G_THF_ (%)	G_Toluene_ (%)	** S_DMF_ (%)	S_THF_ (%)	S_Toluene_ (%)	ρ_0_ (g/cm^−3^)	V_es_ (mol/m^3^)
WPU	−	−	−	−	−	−	1.0499	−
WPU-CO1	−	−	86.5 ± 1.6	−	−	200.9 ± 6.7	1.0521	260.1 ± 17.2
WPU-CO2	−	−	88.2 ± 1	−	−	173.5 ± 5.4	1.0762	332 ± 20.4
WPU-MA	49.4 ± 1.6	53.1 ± 1.8	91.9 ± 0.7	205.4 ± 6.8	156.8 ± 5.3	63.5 ± 2.2	1.0844	2001 ± 110
WPU-TA	49.8 ± 1.5	54.1 ± 1.4	92.1 ± 0.7	185.6 ± 5.7	103.2 ± 3.7	57.7 ± 2	1.1264	2189 ± 117
WPU-CO1-TA	57.1 ± 1.3	70.9 ± 1.2	93.8 ± 0.5	184.6 ± 5	101.4 ± 3.3	58.8 ± 1.7	1.1249	2230 ± 99
WPU-CO2-TA	59.9 ± 1.1	62.1 ± 1.2	95.1 ± 0.5	178.9 ± 4.4	88.9 ± 2.9	52.1 ± 1.5	1.1008	2652 ± 115

* G is gel fraction; ** S is swelling ratio. Although swelling and gel fraction in THF and DMF are presented for comparison, only the toluene data were used for the discussion of network integrity and for the calculation of the apparent effective network density based on the Flory–Rehner equation.

**Table 3:** Mechanical and thermal property parameters of the synthesized WPU films.

Sample	Stress (MPa)	Elongation (%)	Td_10%_ (°C)	Td_50%_ (°C)	Hardness
WPU	2.7	619.3	255.5	333.8	2H
WPU-CO1	23.1	463.9	260.7	348.9	4H
WPU-CO2	25.3	337.9	260.5	346.6	4H
WPU-MA	10.2	102.8	255.6	353.4	4H
WPU-TA	23.2	221.3	255.9	351.4	5H
WPU-CO1-TA	30.4	325.5	257.8	349.3	5H
WPU-CO2-TA	36.0	288.8	263	339.5	6H

## Data Availability

The original contributions presented in this study are included in the article. Further inquiries can be directed to the corresponding author.

## Appendix A

**Table A1:** Formulations of the synthesized WPU films.

Sample	IPDI (mol)	PCL1000 (mol)	DMBA (mol)	HEMA (mol)	PETA (mol)	CO (mol)	TEA (mol)	R-Value
WPU	12.98	3.564	7.25	0	0	−	7.25	1.2
WPU-CO1	13.65	3.564	7.25	0	0	0.37	7.25	1.2
WPU-CO2	14.39	3.564	7.25	0	0	0.79	7.25	1.2
WPU-MA	14.39	3.564	7.25	2.34	0	0	7.25	1.2
WPU-TA	14.39	3.564	7.25	0	2.34	0	7.25	1.2
WPU-CO1-TA	15.05	3.564	7.25	0	2.34	0.37	7.25	1.2
WPU-CO2-TA	15.79	3.564	7.25	0	2.34	0.78	7.25	1.2
